# The social inefficiency of regulating indirect land use change due to biofuels

**DOI:** 10.1038/ncomms15513

**Published:** 2017-06-26

**Authors:** Madhu Khanna, Weiwei Wang, Tara W. Hudiburg, Evan H. DeLucia

**Affiliations:** 1 Department of Agricultural and Consumer Economics, Institute for Sustainability, Energy, and Environment, University of Illinois, Urbana, Illinois 61801, USA; 2 Department of Agricultural and Consumer Economics, University of Illinois, Urbana, Illinois 61801, USA; 3 Department of Forest, Rangeland, and Fire Sciences, 875 Perimeter Drive, University of Idaho, Moscow, Idaho 83863, USA; 4 Department of Plant Biology, Institute for Sustainability, Energy, and Environment, University of Illinois, Urbana, Illinois 61801, USA

## Abstract

Efforts to reduce the indirect land use change (ILUC) -related carbon emissions caused by biofuels has led to inclusion of an ILUC factor as a part of the carbon intensity of biofuels in a Low Carbon Fuel Standard. While previous research has provided varying estimates of this ILUC factor, there has been no research examining the economic effects and additional carbon savings from including this factor in implementing a Low Carbon Fuel Standard. Here we show that inclusion of an ILUC factor in a national Low Carbon Fuel Standard led to additional abatement of cumulative emissions over 2007–2027 by 1.3 to 2.6% (0.6–1.1 billion mega-grams carbon-dioxide-equivalent (Mg CO_2_e^−1^) compared to those without an ILUC factor, depending on the ILUC factors utilized. The welfare cost to the US of this additional abatement ranged from $61 to $187 Mg CO_2_e^−1^ and was substantially greater than the social cost of carbon of $50 Mg CO_2_e^−1^.

## Abstract (web summary)

A Low Carbon Fuel Standard seeks to regulate indirect land use change by including its related carbon emissions in the carbon intensity of biofuels. Khanna *et al*. show the economic cost of abatement achieved by including this factor is much larger than the social cost of carbon.

Low carbon fuel policies at the federal and state level in the US such as the Renewable Fuel Standard (RFS) and the Low Carbon Fuel Standard (LCFS) in California seek to reduce dependence on fossil fuels and carbon emissions by inducing a switch towards biofuels. The RFS sets a quantity mandate for different types of biofuels that differ in their carbon intensity relative to gasoline. The RFS is implemented as a mandate to blend a certain share of biofuels with gasoline annually since 2007. On the other hand, a LCFS sets a target for the percentage reduction in the average carbon intensity of transportation fuel below a baseline level and provides blenders the flexibility to select the mix and quantities of different biofuels to meet the average fuel carbon intensity standard.

The production of biofuels has raised concerns about their competition for land with food crops resulting in higher global crop prices^1^,^2^ that lead to indirect land use change (ILUC) globally by creating incentives for the conversion of non-cropland to crop production and releasing carbon stored in soils and vegetation^3^. Studies differ in their estimate of the extent to which biofuels have affected food crop prices with many studies estimating these to be 14–35% higher than in the baseline depending on the specifics of the biofuel policies, the definition of the baseline, the time frame for the comparison, types of biofuels included and the models and methods utilized^1^,^4^,^5^.

To reduce the potential for ILUC offsetting at least a part of the carbon savings generated by displacing fossil fuels with biofuels, legislation establishing the RFS and the California LCFS require inclusion of the direct- and ILUC-related carbon intensity of a biofuel in determining its total carbon intensity for compliance with these regulations^6^,^7^. The ILUC-related carbon intensity is biofuel-specific and is referred to as the ‘ILUC factor’ of that biofuel. The ILUC factor is a measure of the carbon emissions released per unit of biofuel, due to land use change domestically and internationally caused by the biofuel-induced changes in food/feed crop prices and land rents in the US. It is feedstock-specific and higher for feedstocks that require greater diversion of productive cropland from food crop production to biofuel production than for energy crops that can be grown productively on low-quality soils^8^. The inclusion of the ILUC-related carbon intensity of a biofuel in the carbon intensity of a biofuel for compliance with the LCFS is intended to lead to internalization of these indirect effects and create incentives to shift the mix of biofuels towards those with low ILUC effects, thereby increasing the abatement of global carbon emissions. However, this approach and the ILUC factors used for the California LCFS have been controversial and the subject of lawsuits by biofuel producers^9^.

There is a large literature assessing the magnitude of the ILUC effect of corn ethanol using global equilibrium models^8^. A few studies have also estimated the ILUC effect of cellulosic biofuels from various feedstocks^10^,^11^. Several studies have examined the effect on carbon emissions of various biofuel policies, including the RFS^4^,^12^,^13^,^14^,^15^,^16^, volumetric tax credits^13^,^14^ and a national LCFS^12^,^17^. Others have examined the land use effect of the RFS^4^,^12^,^13^ and a national LCFS in the US^17^ and internationally^18^. None of these studies examined the economic effects and emissions implications of including an ILUC factor when implementing a LCFS^7^,^8^,^19^,^20^,^21^.

For this study, we used an integrated modelling approach^14^ to analyse the effects on economics and carbon emissions of supplementing the RFS with a national LCFS and the implications of implementing the LCFS with and without an ILUC factor over the 2007–2027 period. We combined the Biofuel and Environmental Policy Analysis Model (BEPAM-F)^14^,^22^, with DayCent^23^,^24^,^25^,^26^ to estimate soil carbon sequestration and with the Greenhouse Gases, Regulated Emissions, and Energy Use in Transportation (GREET) model to estimate above-ground life cycle emissions. BEPAM-F is a dynamic, open economy, integrated model of the agricultural, forestry and transportation sectors in the US. DayCent is a globally validated ecosystem model which simulates the direct effects of land use change on soil carbon sequestration and nitrogen cycling. We used this to estimate the spatially heterogeneous feedstock-specific direct life cycle carbon emissions intensity of biofuels together with parameters from GREET^27^. We included feedstock-specific ILUC factor estimates from California Air Resources Board (CARB)^28^, Environmental Protection Agency (EPA)^29^ and Searchinger^10^. Our results show that inclusion of an ILUC factor in a national LCFS leads to additional abatement of cumulative emissions over 2007–2027 by 1.3 to 2.6% (0.6–1.1 Billion Mg CO_2_e^−1^) compared to those without an ILUC factor, depending on the ILUC factors utilized. However, this abatement is achieved at a welfare cost to the US ranging from $61 to $187 Mg CO_2_e^−1^, which is substantially greater than the social cost of carbon of $50 Mg CO_2_e^−1^.

## Results

### Simulated scenarios

The baseline scenario (No_LCFS) was defined as one in which only the RFS is implemented over the 2007–2027 period^12^ (Supplementary Fig. 1). We then supplemented the baseline with two alternative LCFS scenarios, defined as ‘with’ and ‘without’ the inclusion of the ILUC factor in the carbon intensity of biofuels. Both LCFS scenarios set the same targets for reducing the average carbon intensity of fuel over the 2017–2027 period. In the ‘without’ scenario (LCFS_No_ILUC factor), we considered only the direct life cycle carbon intensity of a biofuel (including the carbon intensity due to direct land use change) to determine compliance with the LCFS. In the ‘with’ scenario (LCFS_With_ILUC factor) the sum of the ILUC factor and the direct life cycle GHG intensity of a biofuel was considered.

Studies differ widely in their estimates of the ILUC factor of a feedstock; with estimates ranging from 13 to 104 g CO_2_e Mega-Joule (MJ)^−1^ for corn ethanol and from 5.8–111 g CO_2_e MJ^−1^ for cellulosic biofuels^11^ (Supplementary Table 1), depending on choice of model^8^,^19^,^20^ and underlying assumptions^8^,^11^,^12^,^19^. The first study to quantify the ILUC effect by Searchinger *et al*.^10^ obtained the largest values for the ILUC factor in this range for both corn ethanol and cellulosic ethanol. These large estimates have been shown to result from a number of restrictive assumptions in the modelling analysis including those about the rate of growth of crop productivity, the availability of idle land for conversion to crop production and the ease of conversion of land from one use to another as discussed in Khanna and Crago^8^ and Dumortier *et al*.^30^. Subsequent estimates obtained using alternative modelling approaches by the EPA^29^ for implementing the RFS and by the CARB^31^ for implementing the LCFS were substantially lower due to differences in the model structure and parametric assumptions^8^,^32^.

We considered three cases of the LCFS_With_ILUC factor scenario, using feedstock-specific ILUC factors from CARB^28^, EPA^29^ and Searchinger^10^. Since no study has estimated ILUC factors for all feedstocks considered here, we imputed values from other studies as shown in Supplementary Table 2. Estimates by Searchinger^10^ were included in the spectrum of ILUC factors considered here, despite their limitations, to analyse and illustrate the economic and carbon emission consequences of these extremely large ILUC factors in implementing a LCFS.

The RFS and LCFS policy targets varied over time, thus, the mix and quantities of biofuels and fuels and their economic and carbon emission effects differed over time during the 2007–2027 period. To account for the complete effect of these policies over time, we compared the cumulative ‘global’ emissions between different scenarios. Cumulative emissions were defined as the sum of the direct emissions from the agricultural, forestry and transportation sectors in the US and the ILUC-related emissions from biofuels over the 2007–2027 period. We used the feedstock-specific ILUC factors for estimating the cumulative ILUC-related emissions in each of the three cases of the LCFS_With_ILUC factor scenario. We estimated the change in cumulative emissions in each of the three cases of the LCFS_With_ILUC factor scenario relative to the LCFS_No_ILUC_factor scenario and relative to the No_LCFS scenario. A comparison of the cumulative emissions in each of the three cases of the LCFS_With_ILUC factor scenario with those under the LCFS_No_ILUC factor scenario provided an assessment of the additional abatement achieved globally due to the inclusion of the ILUC factor in each case.

To estimate the economic effects of this abatement, we measured the change in present value of social welfare, defined by the discounted sum of the changes in consumer, producer and government surplus across the agricultural, forestry and transportation sectors over the 2007–2027 period in each of the three cases of the LCFS_With_ILUC scenario relative to the LCFS_No_ILUC factor scenario. We divided the estimate of the difference in economic surplus between the ‘with’ and ‘without’ scenarios, over the 2007–2027 period, by the additional cumulative emissions abated in each of the three cases to obtain a case-specific estimate of the cost of this additional abatement. We compared this cost of abatement to the average social cost of carbon^33^ which is a measure of the monetary value of the damages due to carbon emissions to determine if the ILUC factor approach resulted in a positive or negative net societal benefit.

### Implicit taxes and subsidies under the LCFS

**Figure 1:**
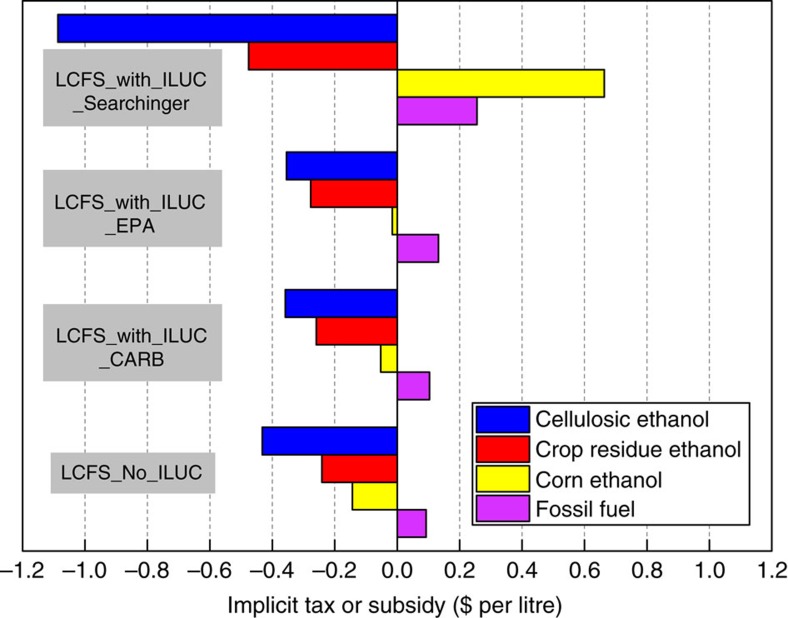
The implicit fuel taxes or subsidies in US dollars per litre with and without ILUC. ILUC refers to indirect land use change. The positive values represent a tax while negative values represent a subsidy on the fuel. Cellulosic ethanol (blue) includes ethanol produced from cellulosic biomass from miscanthus, switchgrass and other perennial energy crop feedstocks, crop residue ethanol (red) is produced from cellulosic biomass in corn and wheat residues, corn ethanol (yellow) is from corn grain and fossil fuels (purple) include gasoline and diesel. CARB, California Air Resources Board; EPA, Environmental Protection Agency, Searchinger, T. *et al*.^10^

The RFS and the LCFS policies implicitly tax gasoline and diesel and subsidize biofuels^12^,^34^. Unlike the RFS, the implicit tax on fossil fuels and implicit subsidy on low carbon biofuels is based on their carbon intensities. These implicit taxes and subsidies are determined by an implicit price per unit of carbon that is the same for all fuels and depends on the stringency of the LCFS target relative to the baseline and by a fuel-specific difference between the fuel’s carbon intensity and the target for average fuel carbon intensity set by the LCFS. Fuels with carbon intensity higher than the standard are implicitly taxed (such as fossil fuels) while those with carbon intensity lower than the standard (such as biofuels) are implicitly subsidized. The inclusion of the ILUC factor in the carbon intensity of biofuels increases the difficulty and thus the implicit price of carbon for achieving a given LCFS target by making biofuels more carbon intensive (Fig. 1). This increases the implicit tax on fossil fuels and creates greater incentives to reduce their consumption. The inclusion of the ILUC factor also reduces the difference between the carbon intensity of a biofuel and the LCFS target. The impact of this on the implicit subsidy for a biofuel is ambiguous and will differ across biofuels; it will increase the implicit subsidy for biofuels with low ILUC factors and reduce the implicit subsidy (or even implicitly tax) biofuels with high ILUC factors. This will thereby induce a shift from biofuels with relatively high ILUC factors towards biofuels with low ILUC factors.

**Figure 2:**
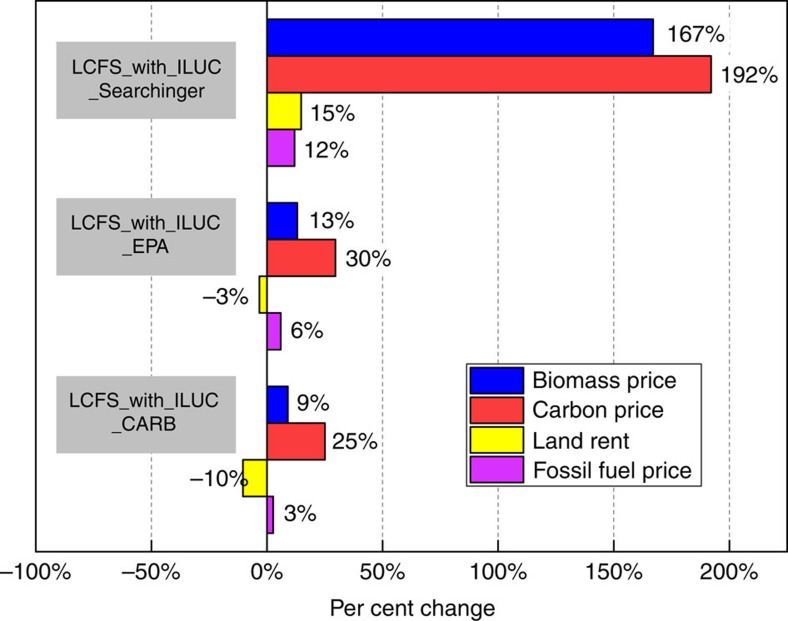
Percentage change in various prices due to inclusion of the ILUC factor. ILUC refers to indirect land use change. Fossil fuel price (purple) is the weighted average price of gasoline and diesel fuel. Carbon price (red) is the implicit price of carbon needed to achieve the LCFS constraint. It is $81 Mg CO_2_e^−1^ in the LCFS with no ILUC factor scenario. Biomass price (blue) is the marginal cost of producing the last unit of biomass to meet the policy induced demand. It is $79 Mg^−1^ in the LCFS with no ILUC factor scenario. CARB, California Air Resources Board; EPA, Environmental Protection Agency, Searchinger, T. *et al*.^10^

We found the implicit carbon price under the LCFS_No_ILUC scenario to be $81 Mg CO_2_e^−1^. The extent to which the inclusion of the ILUC factor increased this implicit carbon price varied across the three cases considered. The CARB, EPA and Searchinger ILUC factors raised the implicit price of carbon by 25, 30 and 192%, respectively, relative to the LCFS_No_ILUC factor scenario (Fig. 2). This carbon price represents the marginal cost of carbon abatement to meet the LCFS. This is different from the welfare cost of carbon abatement discussed below which is based on the change in economic surplus for consumers, producers and government due to the LCFS with or without the ILUC factor relative the No_LCFS scenario.

This increased the implicit tax per litre on fossil fuels (gasoline and diesel) and lowered the implicit subsidy on corn ethanol and energy crops for cellulosic biofuels (Fig. 1); the high Searchinger ILUC factor for corn ethanol converted the implicit subsidy on corn ethanol under the LCFS_No_ILUC scenario to a tax. All three sets of ILUC factors (particularly the Searchinger factor) increased the implicit subsidy for crop residues due to their negligible ILUC factor (Supplementary Table 1). The Searchinger factors also increased the implicit subsidy for certain energy crops (such as willow, poplar and energy cane that were assumed to have a zero ILUC factor because of regions where they are grown while reducing the implicit subsidy for other energy crops (miscanthus and switchgrass) with very high ILUC factors to zero (see Supplementary Table 2).

### Effects on consumption of alternative fuels and land use

Under the No_LCFS scenario there is 57 billion litres of corn ethanol and 70 billion litres of cellulosic ethanol (of this 47 billion litres are from crop residues, mainly corn stover, and the rest from perennial energy crops) in 2027 (Supplementary Table 3). The implementation of the LCFS_No_ILUC factor increased the implicit subsidies for cellulosic biofuels and increased their volume to 110 billion litres, with most of it produced from cellulosic feedstocks, while reducing the amount of corn ethanol to 19 billion litres.

**Figure 3:**
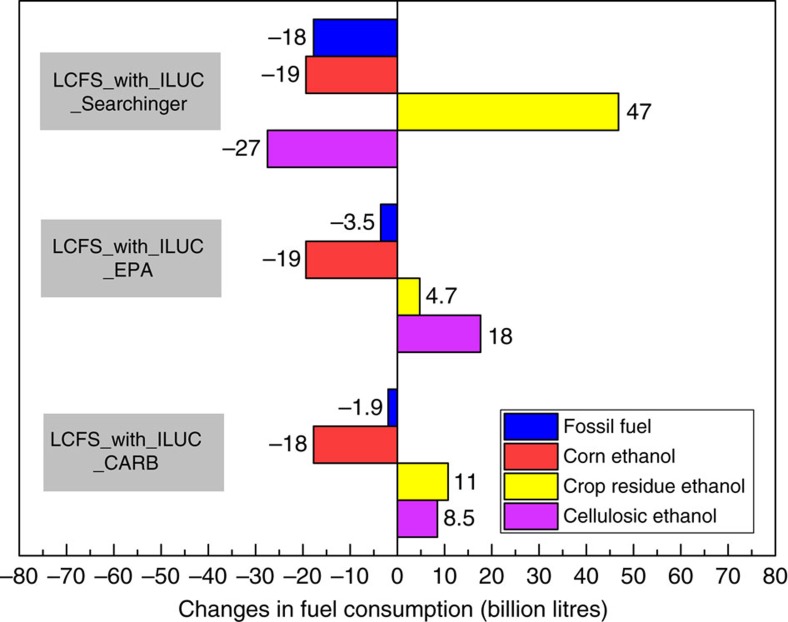
Change in fuel consumption due to inclusion of the ILUC factor in billions of litres. ILUC refers to indirect land use change. Bars show the difference in fuel consumption between the scenario with no ILUC factor and the scenario with ILUC factor. Negative values indicate that fuel use was higher in the scenario with no ILUC factor. Fossil fuel (blue) includes gasoline and diesel; crop residue ethanol (yellow) includes corn stover and wheat straw ethanol; cellulosic ethanol (purple) includes ethanol from perennial energy crops, miscanthus, switchgrass, energy cane, poplar and willow. CARB, California Air Resources Board; EPA, Environmental Protection Agency, Searchinger, T. *et al*.^10^

The addition of an ILUC factor in all three cases (CARB, EPA and Searchinger) reduced the demand for fossil fuels and corn ethanol and increased reliance on cellulosic ethanol; however, the composition of feedstocks for the cellulosic biofuels differed across the three cases. Production of corn ethanol decreased by 18–19 billion litres to levels close to zero in all three cases relative to the LCFS_No_ILUC factor scenario (Fig. 3). The CARB factors led to an 8- and 11-billion-litre increase in cellulosic ethanol from energy crops and crop residue ethanol consumption respectively. The inclusion of the Searchinger ILUC factors reduced perennial grass ethanol from all sources by 27 billion litres (Supplementary Table 3). It also affected the mix of energy crops used to produce ethanol by switching away from those with high ILUC factors such as miscanthus and switchgrass to other perennials, such as energy cane, willow and poplar (Supplementary Table 3). Production of crop residue ethanol increased by 47-billion litres relative to the LCFS_No_ILUC scenario. Despite the assumed ILUC factor for corn stover and wheat straw being the same in all three cases, the larger consumption of corn stover in the Searchinger case was due to limited cost-effective feedstock alternatives with a low ILUC factor. Consequently, this case resulted in a high carbon price and a larger implicit subsidy for crop residues (Figs 2 and 3). The additional demand for cellulosic biofuels in all three cases of the LCFS_With_ILUC factor scenario resulted in a higher price of biomass compared to the $79 Mg^−1^ level in the No_LCFS scenario (Fig. 2). Biomass price increased by 9,13 and 167% under the CARB, EPA and Searchinger cases, respectively.

Under the No_LCFS scenario 13.7 million hectares of land were used in 2027 to produce the corn needed to meet the corn ethanol mandate of 56 billion litres (Supplementary Table 3). This estimate was significantly smaller than the 60 million hectares estimated in Chakravorty *et al*.^4^ because they assumed that the lowest quality cropland (with a yield of 1.7 Mg per hectare) would be used for producing corn for ethanol. We assumed that average quality land with a yield of 10.3 Mg per hectare would be used for corn for ethanol production in 2027. EIA estimates for land used to produce 14.2 billion gallons in 2014 indicate a yield of 9.8 Mg per hectare^35^, while USDA estimates of corn yields in 2015 are 10.6 Mg per hectare^36^. Our findings were similar to those in Hertel *et al*.^37^ who found that 15 million hectares of land would be used for corn for ethanol assuming a 2001 corn yield of 8.5 Mg per hectare. Our findings were also similar to Chen *et al*.^12^ who found that 11.6 million hectares of land would be used to produce 15 billion gallons of corn ethanol in 2030. We also found that 4.2 million hectares of land would be needed to produce 18.8 billion gallons (70 billion litres) of cellulosic ethanol (from all feedstocks) including crop residues in the No_LCFS scenario. This was close to the 4.2 million hectares of land needed to produce 16 billion gallons of cellulosic biofuel in 2030 in Hudiburg *et al*.^14^ but much smaller than the 11 million hectares required to produce 21 billion gallons in Chakravorty *et al*.^4^. This was largely because Chakravorty *et al*.^4^ did not consider the potential to produce biofuels from crop residues which requires no diversion of land.

The implementation of the LCFS ‘with’ and ‘without’ the ILUC factors resulted in a change in land use relative to the No_LCFS scenario (Supplementary Table 1). The LCFS_No_ILUC factor resulted in a reduction in demand for corn ethanol and a shift towards energy crops. Land under corn for ethanol declined to 4.6 million hectares while that under energy crops for cellulosic biofuels increased to 23 million hectares (Supplementary Table 4). The LCFS_With_ILUC factor further exacerbated this shift away from corn ethanol to cellulosic biofuels. Land under energy crops increased to 24–30 million hectares under the three cases of the LCFS_With_ILUC factor scenario. As a result, land under crop production for food, feed and fibre was marginally higher in the LCFS_With_ILUC factor scenario in the CARB and EPA cases.

### Carbon emissions and welfare effects

The estimated US carbon emissions (including those due to ILUC) ranged between 44 and 46.2 B Mg CO_2_e in the No_LCFS scenario across the three sets of ILUC factors (Table 1). These declined by 1.9% (=(43.2–44.0)/44)) to 5.1% (=(43.9–46.2)/46.2)) in the LCFS_No_ILUC factor scenario relative to the No_LCFS scenario (percentage estimates are before rounding off of carbon emission estimates). The largest decline was observed in the Searchinger case and occurred primarily because of the high baseline emissions in this case in the No_LCFS scenario due to the high ILUC factor for corn ethanol. The implementation of the LCFS_With_ILUC factor led to an additional abatement of 1.3–2.6% relative to the LCFS_No_ILUC factor scenario. This amounted to 0.6–1.1 B Mg CO_2_e across the three cases.

The LCFS_No_ILUC policy increased the economic surplus of food and fuel consumers while adversely affecting fossil fuel producers. There was a small net increase in the discounted value of cumulative economic surplus (2007–2027) by $35 billion relative to the No_LCFS baseline (by 0.13%), assuming a 3% discount rate (Table 1). This was different from the result obtained in Huang *et al*.^17^ which showed a slight decline (0.17%) in social welfare in the LCFS_No_ILUC scenario relative to the No_LCFS scenario. This was due to higher values for the direct carbon intensities of energy crop feedstocks assumed in that study that were based on previous literature. The carbon intensity of energy crops assumed here were based on a calibrated and validated DayCent model and were significantly lower, resulting in lower costs of implementing the LCFS. Chen *et al*.^12^ found that a national LCFS implemented by itself would lead to an increase in US social welfare by $33.4 B over the 2007–2030 period relative to a no-policy scenario.

**Figure 4:**
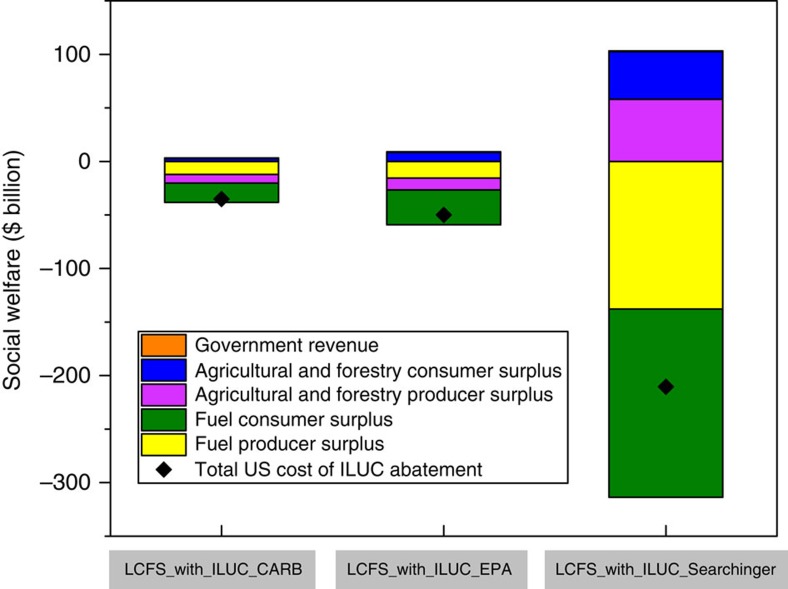
Effects of each ILUC factor on the discounted value of economic surplus in billions of dollars relative to a scenario without this factor. ILUC refers to indirect land use change. Bars show the difference in economic surplus between the scenario with no ILUC factor and the scenario with ILUC factor. Negative values indicate a net cost because the surplus was higher in the scenario with no ILUC factor. Surpluses are divided into five categories: government revenue (orange), agricultural and forestry consumer (blue), agricultural and forest producer (purple), fuel consumer (green) and fuel producer (yellow). The total US cost of abatement by including the ILUC factor is represented by the dark blue diamond; this net cost is the sum of the change in discounted value of the economic surplus of consumers and producers in the agricultural, forestry and transportation sectors and the government surplus over the 2007–2027 period between the LCFS with ILUC factor and LCFS with no ILUC factor scenarios; change in government surplus was negligible. These net costs are $35.1 billion with CARB factors, $50 billion with EPA factors and $211 billion with Searchinger factors. CARB, California Air Resources Board; EPA, Environmental Protection Agency, Searchinger, T. *et al*.^10^

The additional cost of implementing the LCFS was estimated as the difference in discounted social welfare between the LCFS_No_ILUC and LCFS_With_ILUC scenarios. As compared to the LCFS_No_ILUC scenario, the higher implicit tax on fossil fuels and the lower implicit subsidy on biofuels increased the price of fuel for consumers and lowered the price received by agricultural and fuel producers; the net loss in economic surplus for producers ranged from $20 to $80 billion (Fig. 4 and Supplementary Table 4). The net reduction in total consumer surplus ranged from $15 to $131 billion. These losses were largest with the Searchinger factors. The overall reduction in social welfare for consumers, producers and government across the sectors considered here ranged between $35 and $211 B. It was highest with the Searchinger factors and lowest with the CARB factors. Over half of this loss in economic surplus was borne by the fuel consumers (Fig. 4 and Supplementary Table 4).

We divided the additional cost by the additional abatement achieved with the inclusion of the ILUC factor (0.6–1.1 B Mg CO_2_e) to obtain the per metric ton welfare cost of abatement. We found this ranged from $61 Mg CO_2_e^−1^ (=$35B/0.6 B Mg CO_2_e) to $187 Mg CO_2_e^−1^ (=$211B/1.1 B Mg CO_2_e). This welfare cost of abatement per metric ton is lower than the marginal cost of abatement implied by the price of carbon discussed earlier which was $81 Mg CO_2_e^−1^ in the LCFS_No-ILUC scenario and ranged from $101 to $235 Mg CO_2_e^−1^ in the LCFS_With_ILUC scenarios; the higher end of the range was estimated with the Searchinger factors. Even the welfare cost of abatement per metric ton was substantially higher (20–270%) than the average social cost of carbon of $50 Mg CO_2_e^−1^ with the same 3% discount rate assumed here^33^ (Table 1).

There is wide disparity in the range of estimates of the social cost of carbon^38^ but considerable consensus that $50 Mg CO_2_e^−1^ is a reasonable estimate. Following an extensive review of the estimates of the social cost of carbon in the literature, Tol (2005)^39^ concluded that the social cost of carbon in 2030 was unlikely to exceed $50 Mg CO_2_e^−1^, under standard assumptions about discounting and aggregation. Based on a similar review, Watkiss and Downing (2008)^40^ found that $50 Mg CO_2_e^−1^ provided a reasonable benchmark for global decision making seeking to reduce the threat of dangerous climate change and including a modest level of aversion to extreme risks, relatively low discount rates and equity weighting. Most recently Havranek *et al*. (2015)^41^ conducted a meta-analysis of estimates of the social cost of carbon in the literature and found that the upper boundary for mean estimates of the social cost of carbon reported by studies after controlling for various factors (including publication bias) was $39 Mg CO_2_e^−1^. Estimates of the social cost of carbon have a skewed, right-tailed distribution^33^. This implies a relatively smaller likelihood of their exceeding the cost of abatement estimated here than of being lower than it. Cost of abatement with the Searchinger factors ($187 Mg CO_2_e^−1^) was higher than even the 95th percentile of the social cost of carbon of $152Mg CO_2_e^−1^ with the same 3% discount rate assumed here.

We examined the sensitivity of our findings to several key parameters assumed here by considering alternative values for: the elasticity of supply of gasoline from the rest of the world, feedstock yields, cost of conversion to ethanol and carbon emissions due to conversion of marginal land to cropland (Supplementary Fig. 2). We found that these costs of abatement could increase significantly under more conservative assumptions about the yields and availability of marginal land for perennial grasses and the costs of producing cellulosic biofuels. Cost of abatement ranged between $54 and $94 Mg CO_2_e^−1^ with the CARB factors; corresponding ranges were $63-$107 with the EPA factors and $162-$199 with the Searchinger factors. Lastly, we investigated the sensitivity to the discount rate by increasing it from 3 to 5%. Cost of abatement with a 5% discount rate was $45-$122 Mg CO_2_e^−1^. These costs were 181 to 662% higher than the correspondingly lower average social cost of carbon of $16 Mg CO_2_e^−1^ in 2030 (ref. 33).

## Discussion

Our analysis examined the effectiveness of an ILUC factor approach while implementing a national LCFS in achieving additional reduction in carbon emissions and the welfare costs at which these reductions were achieved. Estimates of the ILUC factor of a biofuel differ considerably across studies. We selected estimates from three different sources to analyse the range in outcomes in response to variability in ILUC estimates. In all cases, we found that the inclusion of the ILUC factor in implementing the LCFS imposed additional costs on fuel consumers and fuel producers because it lowered the implicit subsidy on several types of biofuels while raising the implicit tax on fossil fuels. It led to additional abatement of cumulative emissions over 2007–2027 by 0.6–1.1 B Mg CO_2_e compared to those without an ILUC factor. The relatively higher ILUC factors for both corn ethanol and cellulosic ethanol in the Searchinger case led to a higher implicit carbon price and greater reduction in carbon emissions relative to the other two cases. However, this also imposed very high costs on fuel consumers and producers. The overall discounted welfare cost of abatement over the 2007–2027 period on the agricultural, forestry and transportation sectors considered here ranged from $35 B to $211 B; the largest cost was obtained with the Searchinger factors.

These values implied that the per unit cost of additional abatement to the US ranged from $61 to $187 Mg CO_2_e^−1^. We found that across all three cases of ILUC factors, this cost of abatement was substantially greater than the social cost of carbon of $50 Mg CO_2_e^−1^ in 2030, with the same 3% discount rate used in both cases. A higher discount rate of 5%, lowered the cost of abatement to range between $45 and $122 Mg CO_2_e^−1^ across the three cases of ILUC factors. These costs were 181 to 662% higher than the correspondingly lower average social cost of carbon of $16 Mg CO_2_e^−1^ in 2030. Our analysis, therefore, showed that the ILUC factor approach to reducing ILUC-related carbon emissions with a LCFS did not result in positive net social benefits; the monetary value of the benefits from the additional abatement achieved was lower than the cost of achieving that abatement for the US.

Leakage of carbon emissions due to ILUC is an issue of concern since it offsets the direct savings in emissions due to displacement of fossil fuels by biofuels. Alternatives to the ILUC factor approach include those that directly address the source of the problem, namely, the choice of feedstock and the land on which it is grown, and thereby reduce the potential for indirect market effects^1^,^4^,^7^. Food-crop-based biofuels, like corn ethanol, have a high ILUC effect and also a high direct carbon intensity (Supplementary Table 1). In contrast, cellulosic feedstocks have low direct carbon intensity. Accompanying biofuel policies like the RFS and/or LCFS with an explicit carbon price policy that penalizes fuels based on their direct carbon intensity can provide incentives to switch away from corn ethanol to low carbon cellulosic feedstocks. Alternatively, certification of low indirect impact biofuels and policies that restrict blending of non-certified biofuels can create incentives to produce more low ILUC feedstocks. Direct regulations to restrict conversion of grasslands and forestland to cropland can also prevent indirect loss of carbon. We leave it to future research to examine the cost-effectiveness of such approaches compared to that of an ILUC factor approach.

## Methods

### Economic modelling

BEPAM-F (Biofuel and Environmental Policy Analysis Model with Forestry), is a spatially explicit multi-market dynamic open economy model that determines the market equilibrium by maximizing the sum of consumers’ and producers’ surpluses in the agricultural, forestry and transportation fuel sectors in the US subject to various material balance, technological, land availability, and policy constraints over the 2007–2027 period. The model includes crop, forest and pasture land in the US with the potential for conversion of land from one use to another based on the net returns to land under various uses subject to some constraints. The BEPAM-F integrates the transportation, agriculture and forest sectors to endogenously determine the effects of alternative policy scenarios on land allocation among food and biofuel crops, fuel mix, prices in markets for fossil fuel, biofuel, food/feed crops and livestock and on carbon emissions in the US at 5-year intervals over the period 2007–2027. Model structure, parameterization and validation were explained in previous studies^12^,^14^,^17^.

The transportation sector incorporates downward sloping demand curves for vehicle kilometres travelled (VKT) with four types of vehicles (conventional gasoline, flex fuel, gasoline-hybrid and diesel vehicles) that generate a derived demand for liquid fossil fuels and biofuels that include first- and second-generation biofuels. The VKT production function considers the energy content of alternative fuels, fuel economy of each type of vehicle and the forthcoming Corporate Average Fuel Economy standards, and technological limits on blending gasoline and ethanol for each of these four types of vehicles^42^.Gasoline is produced domestically and imported. Supply curves for domestic gasoline and diesel as well as for gasoline supply and demand in rest of world are included to determine the amount of gasoline imports and the price of gasoline and diesel. Several first- and second-generation biofuels that can be blended with gasoline and diesel were considered (Supplementary Table 2). First-generation biofuels include domestically produced corn ethanol and imported sugarcane ethanol, soybean biodiesel, DDGS-derived corn oil and waste grease. Second-generation biofuels include cellulosic ethanol and biomass-to-liquid diesel that can be blended with gasoline and diesel, respectively. We determined the domestic and international price of gasoline endogenously by the domestic demand for gasoline derived from the downward sloping demands for VKT and the demand for gasoline in the rest of the world and the upward sloping domestic and the rest of the world supply of gasoline. The policy induced increased production of biofuels reduces the domestic demand for gasoline and the US demand for imports from the rest of the world. We incorporated the feedback effect of the biofuel-driven reduction in the world and domestic price of gasoline on fuel consumption in the US and its implications for the carbon savings with biofuels^12^,^43^.

The agricultural and forestry sectors produce a broad range of crop, livestock, bioenergy and forest products that compete for land. The prior application of BEPAM-F^14^ focused on analysing the feedstock mix, land use and GHG implications of a cellulosic biofuel mandate over the 2007–2027 period. The agricultural sector in BEPAM-F includes all major conventional crops and livestock animals, four energy crops (miscanthus, switchgrass, energy cane, hybrid poplar and willow) and two crop residues (corn and wheat) as well as choice of tillage practice and crop rotations for conventional crops. It incorporated spatial heterogeneity in the yields and costs of production of various crops and livestock products, availability of different types of land and costs of conversion of cropland pasture to cropland across the 295 crop-reporting districts (CRDs) in the US. Availability of five types of agricultural land (irrigated and non-irrigated cropland, idle cropland, cropland pasture, and pasture land) were specified for each CRD. Changes in the mix of crops grown were determined using the methods described in Chen and Önal (2012)^44^. Assumptions about the productivity of cropland pasture, the costs of converting it to conventional crops or energy crops, and restrictions on land conversion for energy crop production in a CRD were similar to Hudiburg *et al*.^14^. The structure of the forestry sector in BEPAM-F was similar to that in Forest and Agricultural Sector Optimization Model^16^ and included 11 forest marketing regions. Forestland was characterized by two types of trees (softwoods and hardwoods) and distinguished by various site productivity classes that determined yield per unit land. Land conversion from one use to another within the sector and across sectors was constrained by pre-defined suitability classes that determined which acres could be converted to forest, crop or pasture. A detailed description of forestry sector module in BEPAM-F is provided in Wang *et al*.^22^. Model validation is provided in Wang *et al*.^22^ and in Hudiburg *et al*.^14^.

A key extension of BEPAM-F here is the imposition of a LCFS constraint that restricts the ratio of the sum of the GHG emissions with each type of fossil fuel and biofuel consumed (defined as the sum of the product of the GHG intensity of each fuel and the quantities of those fuels consumed) to the sum of the energy from these fuels to be less than the targeted standard. The GHG intensity of each type of biofuel feedstock included below-ground changes in soil carbon and above-ground emissions. We quantified the major factors influencing the direct life cycle carbon emissions above ground and sequestration below ground due to bioenergy crop production and carbon emissions due to gasoline and diesel consumption in each policy scenario. We simulated the soil organic carbon changes and associated direct N_2_O, CH_4_, NO_3_ leaching for each modelled crop using DayCent^14^,^24^,^26^. DayCent calculates plant growth as a function of water, light, and soil temperature and limits actual growth based on soil nutrient availability. In addition to soil carbon uptake and loss, the DayCent model was also used to simulate harvested yields, direct N_2_O emissions (indirect calculated using IPCC Tier 1 factors), nitrate leaching, and methane flux. Model parameterization, calibration and validation were completed in prior studies^14^,^24^. The direct above-ground life cycle GHG intensity of each of the biofuel pathways was estimated by adapting the Greenhouse Gases, Regulated Emissions, and Energy Use in Transportation (GREET) model as in Dwivedi *et al*.^27^. Both the below-ground and above-ground emissions vary spatially. As a result, the GHG intensity of the overall transportation fuel depended on the mix of feedstocks and the spatial location where they were produced.

Emissions due to ILUC both domestic and globally were included through the ILUC factors. The ILUC factors assumed in this paper were estimated using global economic models that provide estimates of the carbon emissions due to land use change in the US and rest of the world caused by biofuel production. Specifically, ILUC factors estimated by CARB^32^ are obtained from the Global Trade Analysis Project (GTAP) model that is a global general equilibrium model. ILUC factors estimated by Searchinger *et al*.^10^ and by EPA^29^ were estimated using the global partial equilibrium, Food and Agricultural Policy Research Institute (FAPRI) model. These models are described in greater detail in Khanna *et al*.^32^. The estimation of ILUC factors is sensitive to a number of modelling and parametric assumptions as discussed in Khanna and Crago^8^. By adding the ILUC factors to the direct carbon intensity of feedstocks in the US and comparing emissions across scenarios we obtain the change in global emissions in the various policy scenarios.

With the LCFS constraint, the model endogenously determined the mix of feedstock, the locations to grow them in taking into account the spatially varying direct GHG intensity of biofuels, the implicit price of carbon, the fuel-specific implicit taxes/subsidies. In each of the scenarios, we examined the cumulative change (summed over 2007–2027) in global GHG emissions which was the sum of the emissions from the US transportation, agricultural and forestry sectors (including the direct emissions from biofuel production and soil carbon sequestration) and the emissions due to the scenario-specific ILUC effect in the rest of the world due to biofuels. The three policy scenarios simulated here are one with no LCFS baseline (No_LCFS), a second LCFS with no ILUC factor (LCFS_No_ILUC) and a third LFCS with ILUC factor (LCFS_With_ILUC). In the no LCFS baseline a mandated level of biofuel production based on the RFS established by EISA, 2007 was imposed as a blend mandate as in ref. 12 (Supplementary Fig. 1). Unlike the RFS which mandated blending of 36 billion gallon (136.3 billion litres) of ethanol with gasoline by 2022 (considered earlier in ref. 24) with an implicit upper limit of 15 billion gallons on corn ethanol, we imposed a lower mandate of 35 billion gallons (131.5 billion litres) by 2027 with a maximum of 15 billion gallons of corn ethanol, assuming the remaining volumes could be met by sources not included in the model such as municipal solid waste, animal fats and waste oil. Sugarcane ethanol imports from Brazil were allowed with the level determined endogenously based on competitiveness with corn ethanol and cellulosic ethanol, up to a maximum of 4 billion gallons.

In the LCFS with no ILUC factor scenario the RFS in Scenario 1 was supplemented by a LCFS imposed in 2017 to achieve a targeted reduction in average fuel carbon intensity of 15% by 2027 relative to the level in 2007. The GHG intensity of biofuels here included only the direct life cycle emissions. The LCFS with LUC factor scenario is the same as above, except that the GHG intensity of biofuels included both the direct life cycle emissions and ILUC-related emissions intensity obtained from three existing studies, CARB^28^, EPA^29^, and Searchinger^10^.

### Ecosystem modelling

Required inputs for the model include vegetation cover, daily precipitation and temperature, soil texture and current and historical land use practices. Soil organic carbon is estimated from the turnover of soil organic matter pools, which change with the decomposition rate of dead plant material. For the perennial grasses, crop specific physiological parameterizations were performed using the values from a synthesis of studies. We simulated county-level yields for corn grain and stover removals, soy, miscanthus and switchgrass in the US on cropland and marginal land. We define marginal land as land that has been historically less productive cropland and has been idle or set aside as pasture for grazing. Daily climate data were downloaded from the Daymet database (http://daymet.ornl.gov/;1). Historical simulations on cropland followed native vegetation (for example, grasslands) with disturbance history (for example, fire and harvest) followed by ∼110 years of agricultural history. Agricultural history included corn–soy rotations, alfalfa and wheat. Soil carbon stocks were simulated to represent the pre-agricultural native vegetation levels with a subsequent decline as the land was cultivated each year for the annual crops. Model output of yield and soil carbon were evaluated against data at a variety of scales and further evaluation of direct N_2_O were compared with observations in Hudiburg *et al*.^24^. Indirect N_2_O emissions were calculated using the IPCC indirect emission factor for leaching/runoff (0.75%) and the IPCC indirect emission factor for volatilized N (1%). DayCent modelled CH_4_ emissions (consumption through oxidation in non-flooded soils) have been evaluated in US cropping systems^45^. Moreover, DayCent output of crop yields and carbon emissions has been evaluated in numerous studies and at sites all around the world^25^,^26^,^46^,^47^,^48^,^49^,^50^.

### Sensitivity analysis

There is uncertainty about several key parameters assumed in our modelling framework. We examined the sensitivity of our results to alternative values of these parameters. Specifically, our benchmark analysis assumed a fairly inelastic supply of gasoline in the rest of the world (elasticity 0.2)^12^. With an inelastic supply of gasoline, the displacement of demand for gasoline due to the production of biofuels in the US results in a large reduction in the world price of oil which lowers the effects of the implicit tax on fossil fuels imposed by the LCFS on fuel consumers. Thus the effect of the ILUC factor on fuel consumers is mitigated and the cost of abatement is lower. We examined the sensitivity of our results to two extreme assumptions about the elasticity of supply of gasoline: a very elastic supply of gasoline (elasticity of 20) and a very inelastic supply of gasoline (elasticity of 0.1). The economic cost of the LCFS will be higher the higher the cost of cellulosic biofuels. In particular, the industrial cost of conversion of feedstock to cellulosic biofuel is uncertain in the absence of significant commercial scale production. We considered the effects of this cost being 50% higher than in the benchmark. The yield per acre of energy crops assumed in the model is based on simulated results from DayCent; these yields could be lower in practice. We examined the effects of these yields being 25% lower than the benchmark level. We also considered the effects of including emissions from the conversion of cropland pasture to cropland (1.85 MgCO2/ha/yr) as suggested by some studies^11^.

### Code availability

The size and complexity of the code preclude its online availability. However, it is available on request from the authors.

### Data availability

The data needed for replication of results is available on request from the authors.

## Additional information

**How to cite this article:** Khanna, M. *et al*. The social inefficiency of regulating indirect land use change due to biofuels. *Nat. Commun.* **8**, 15513 doi: 10.1038/ncomms15513 (2017).

**Publisher’s note:** Springer Nature remains neutral with regard to jurisdictional claims in published maps and institutional affiliations.

## Supplementary Material

Supplementary Information

## Figures and Tables

**Table 1:** Cumulative carbon emissions and social welfare under alternative scenarios (2007–2027).

**Scenario**	**No_LCFS baseline**			**LCFS_No_ILUC factor**			**LCFS_With_ILUC factor**		
	**CARB**	**EPA**	**Searchinger**	**CARB**	**EPA**	**Searchinger**	**CARB**	**EPA**	**Searchinger**
1. US direct GHG emissions (B Mg CO_2_e)	43.5			42.7			42.2	42.1	41.8
2. US GHG emissions (incl. ILUC) (B Mg CO_2_e)	44.0	44.2	46.2	43.2	43.5	43.9	42.6	42.8	42.7
3. Percentage change in emissions relative to No_LCFS scenario*				−1.9%	−1.7%	−5.1%	−3.2%	−3.3%	−7.6%
4. Change in emissions with ILUC factor relative to LCFS_ No_ILUC scenario† (B Mg CO_2_e)							0.6	0.7	1.1
5.Percentage change in emissions with ILUC factor relative to LCFS_No_ILUC scenario‡							−1.3%	−1.6%	−2.6%
6.US social welfare ($B)	27,514			27,549			27,513	27,498	27,338
7.Social welfare cost of LCFS ($ B)				−35§			0.2||	15||	176||
8.Additional US abatement cost due to ILUC ($ B)¶							35	50	211
9.Additional US cost of global abatement due to ILUC ($/Mg CO_2_e)#							60.7	73.7	186.6

CARB, California Air Resources Board; EPA, Environmental Protection Agency; LCFS, Low Carbon Fuel Standard.

^*^(Emissions with LCFS–Emissions with No_LCFS)/Emissions with No_LCFS.

^†^Emissions with LCFS_No_ILUC minus Emissions with LCFS_With_ILUC.

^‡^(Emissions with LCFS_With_ILUC minus Emissions with LCFS_No_ILUC)/Emissions with LCFS_NO_ILUC.

^§^Social Welfare with No_LCFS minus Social Welfare with LCFS_No_ILUC; negative value indicates a welfare gain with LCFS relative to No LCFS.

^||^Social Welfare with No_LCFS minus Social Welfare with LCFS_With_ILUC; positive value indicates a loss in welfare due to the addition of the ILUC factor relative to the LCFS_No_ILUC scenario.

^¶^Social Welfare with LCFS_No_ILUC factor minus Social Welfare in LCFS_With_ILUC factor; positive value indicates a loss in social welfare due to the addition of the ILUC factor relative to the LCFS_No_ILUC factor.

^#^‘Additional US Abatement Cost Due to ILUC’ divided by ‘Change in Emissions with ILUC Relative to No_ILUC Scenario’ (Row9=Row 8/Row 4).

## References

[b1] Roberts M. J. & Schlenker W. Identifying supply and demand elasticities of agricultural commodities: implications for the US ethanol mandate. Am. Econ. Rev. 103, 2265–2295 (2013).

[b2] Zilberman D., Hochman G., Rajagopal D., Sexton S. & Timilsina G. The impact of biofuels on commodity food prices: assessment of findings. Am. J. Agric. Econ. 95, 275–281 (2013).

[b3] Anderson-Teixeira K. J. . Climate-regulation Services of Natural and Agricultural Ecoregions of the Americas. Nat. Clim. Change 2, 177–181 (2012).

[b4] Chakravorty U., Hubert M.-H., Moreaux M. & Nøstbakken L. The long run impact of biofuels on food prices. Scand. J. Econ. doi:; DOI: 10.1111/sjoe.12177 (2016).

[b5] Chen X. & Khanna M. Food versus fuel: the effect of biofuel policies. Am. J. Agric. Econ. 95, 289–295 (2013).

[b6] Rajagopal D. Consequential life cycle assessment of policy vulnerability to price effects. J. Ind. Ecol. 18, 164–175 (2014).

[b7] Witcover J., Yeh S. & Sperling D. Policy options to address global land use change from biofuels. Energy Policy 56, 63–74 (2013).

[b8] Khanna M. & Crago C. L. Measuring indirect land use change with biofuels: implications for policy. Annu. Rev. Resour. Econ. 4, 161–184 (2012).

[b9] Bevill K. Carbon Counter: California’s Air Resources Board Defends and Reconsiders its Low Carbon Fuel Standard. *Ethanol Producer Magazine*, http://www.ethanolproducer.com/articles/7483/carbon-counters (2011).

[b10] Searchinger T. . Use of U.S. croplands for biofuels increases greenhouse gases through emissions from land-use change. Science 319, 1238–1240 (2008). 18258860 10.1126/science.1151861

[b11] Taheripour F. & Tyner W. E. Induced land use emissions due to first and second generation biofuels and uncertainty in land use emission factors. Econ. Res. Int. 2013, 1–12 (2013).

[b12] Chen X., Huang H., Khanna M. & Önal H. Alternative transportation fuel standards: welfare effects and climate benefits. J. Environ. Econ. Manag. 67, 241–257 (2014).

[b13] Chen X., Huang H. & Khanna M. Land-use and greenhouse gas implications of biofuels: role of technology and policy. Clim. Change Econ. 03, 1250013 (2012).

[b14] Hudiburg T. W. . Impacts of a 32-billion-gallon bioenergy landscape on land and fossil fuel use in the US. Nat. Energy 1, 15005 (2016).

[b15] Bento A. M., Klotz R. & Landry J. R. Are there carbon savings from US biofuel policies? the critical importance of accounting for leakage in land and fuel markets. Energy J. 36, 75–109 (2015).

[b16] Beach R. H. & McCarl B. A. U.S. Agricultural and Forestry Impacts of the Energy Independence and Security Act: FASOM Results and Model Description Final Report Prepared for US Environmental Protection Agency (2010).

[b17] Huang H., Khanna M., Önal H. & Chen X. Stacking low carbon policies on the renewable fuels standard: economic and greenhouse gas implications. Energy Policy 56, 5–15 (2013).

[b18] Msangi S., Batka M., Yeh S. & Witcover J. Analysis of iLUC Impacts Under LCFS Policy: Exploring Impact Pathways and Mitigation Options National LCFS Project, University of California (2012).

[b19] Plevin R. J., Jones A. D., Torn M. S. & Gibbs H. K. Greenhouse gas emissions from biofuels’ indirect land use change are uncertain but may be much greater than previously estimated. Environ. Sci. Technol. 44, 8015–8021 (2010). 20942480 10.1021/es101946t

[b20] Zilberman D., Hochman G. & Rajagopal D. On the inclusion of indirect land use in biofuel regulations. Univ. Ill. Law Rev. 2, 413–433 (2011).

[b21] Zilberman D. Indirect land use change: much ado about (almost) nothing. GCB Bioenergy 9, 485–488 (2016).

[b22] Wang W., Dwivedi P., Abt R. & Khanna M. Carbon savings with transatlantic trade in pellets: accounting for market-driven effects. Environ. Res. Lett. 10, 114019 (2015).

[b23] Parton W. J., Hartman M., Ojima D. & Schimel D. DAYCENT and its land surface submodel: description and testing. Glob. Planet. Change 19, 35–48 (1998).

[b24] Hudiburg T. W., Davis S. C., Parton W. & Delucia E. H. Bioenergy crop greenhouse gas mitigation potential under a range of management practices. GCB Bioenergy 7, 366–374 (2015).

[b25] Campbell E. E. . Assessing the soil carbon, biomass production, and nitrous oxide emission impact of corn stover management for bioenergy feedstock production using DAYCENT. BioEnergy Res. 7, 491–502 (2014).

[b26] Delgrosso S., Mosier A., Parton W. & Ojima D. DAYCENT model analysis of past and contemporary soil NO and net greenhouse gas flux for major crops in the USA. Soil Tillage Res. 83, 9–24 (2005).

[b27] Dwivedi P. . Cost of abating greenhouse gas emissions with cellulosic ethanol. Environ. Sci. Technol. 49, 2512–2522 (2015). 25588032 10.1021/es5052588

[b28] California Air Resources Board. *Staff Report: Initial Statement of* *Reasons for Proposed Rulemaking. Industrial Strategies Division, California Environmental Protection Agency*. Available at: http://www.arb.ca.gov/regact/2015/lcfs2015/lcfs15isor.pdf (2014).

[b29] Environmental Protection Agency. *Renewable Fuel Standard Program (RFS2) Regulatory Impact Analysis.* EPA-420-R-10-006. Washington DC. P. 1107. Available at: https://www.epa.gov/sites/production/files/2015-08/documents/420r10006.pdf (2010).

[b30] Dumortier J. . Sensitivity of carbon emission estimates from indirect land-use change. Appl. Econ. Perspect. Policy 33, 428–448 (2011).

[b31] CARB. Proposed Regulation to Implement the Low Carbon Fuel Standard Volume I. Staff Report: Initial Statement of Reasons P. 374 Available at: https://www.arb.ca.gov/fuels/lcfs/030409lcfs_isor_vol1.pdf California Environmental Protection Agency Air Resouces Board (2009).

[b32] Khanna M., Zilberman D. & Crago C. in *Oxford Handbook of Land Economics* (eds Duke, J.M. & Wu, J.J.) 210–235 (Oxford University Press, Oxford, UK (2014).

[b33] United States Government. *Technical Support Document**: Technical Update of the Social Cost of Carbon for Regulatory Impact Analysis.* Executive Order 12866. Interagency Working Group on Social Cost of Greenhouse Gases, United States Government. P. 35. Available at: https://www.epa.gov/sites/production/files/2016-12/documents/sc_co2_tsd_august_2016.pdf (2013).

[b34] Holland S. P., Hughes J. E., Knittel C. R. & Parker N. C. Unintended consequences of carbon policies: transportation fuels, land-use, emissions, and innovation. Energy J. doi:10.5547/01956574.36.3.2 (2015).

[b35] EIA. Corn Ethanol Yields Continue to Improve (2015). Available at: http://www.eia.gov/todayinenergy/detail.php?id=21212 (2015).

[b36] USDA. Crop Production 2015 Summary (2016). Available at: http://www.usda.gov/nass/PUBS/TODAYRPT/cropan16.pdf.

[b37] Hertel T. W. . Effects of US maize ethanol on global land use and greenhouse gas emissions: estimating market-mediated responses. BioScience 60, 223–231 (2010).

[b38] Yohe G. W., Lasco R. D. & Ahmad Q. K. in Climate Change 2007: Impacts, Adaptation and Vulnerability. Contribution of Working Group II to the Fourth Assessment Report of the Intergovernmental Panel on Climate Change (eds Parry, M.L., Canziani, O.F., Palutik, J.P., van der Linden, P.J. & Hanson, C.E.) 811–841Cambridge University Press, Cambridge, UK (2007).

[b39] Tol R. S. J. The marginal damage costs of carbon dioxide emissions: an assessment of the uncertainties. Energy Policy 33, 2064–2074 (2005).

[b40] Watkiss P. & Downing T. The social cost of carbon: valuation estimates and their use in UK policy. Integr. Assess. 8, 2008 (2008).

[b41] Havranek T., Irsova Z., Janda K. & Zilberman D. Selective reporting and the social cost of carbon. Energy Econ. 51, 394–406 (2015).

[b42] EIA. *Annual Energy Outlook 2010: With Projections to 2035.* Report No. DOE/EIA-0383. Available at: http://www19.iadb.org/intal/intalcdi/pe/2010/05499.pdf (U.S. Energy Information Administration, Office of Integrated Analysis and Forecasting, Washington DC, 2010).

[b43] Chen X. & Khanna M. The market-mediated effects of low carbon fuel policies. AgBioForum 15, 89–105 (2012).

[b44] Chen X. & Önal H. Modeling agricultural supply response using mathematical programming and crop mixes. Am. J. Agric. Econ. 94, 674–686 (2012).

[b45] Del Grosso S. J. . Managing Agricultural Greenhouse Gases: Coordinated Agricultural Research through Gracenet to Address Our Changing Climate 241–250Elsevier (2012). 10.2134/jeq2014.05.0221br 25603267

[b46] Del Grosso S. J. . Global scale DAYCENT model analysis of greenhouse gas emissions and mitigation strategies for cropped soils. Glob. Planet. Change 67, 44–50 (2009).

[b47] Del Grosso S. J., Halvorson A. D. & Parton W. J. Testing DAYCENT model simulations of corn yields and nitrous oxide emissions in irrigated tillage systems in Colorado. J. Environ. Qual. 37, 1383 (2008). 18574169 10.2134/jeq2007.0292

[b48] Cheng K., Ogle S. M., Parton W. J. & Pan G. Simulating greenhouse gas mitigation potentials for Chinese croplands using the DAYCENT ecosystem model. Glob. Change Biol. 20, 948–962 (2014). 10.1111/gcb.12368 23966349

[b49] Cheng K., Ogle S. M., Parton W. J. & Pan G. Predicting methanogenesis from rice paddies using the DAYCENT ecosystem model. Ecol. Model. 261-262, 19–31 (2013).

[b50] Chamberlain J. F., Miller S. A. & Frederick J. R. Using DAYCENT to quantify on-farm GHG emissions and N dynamics of land use conversion to N-managed switchgrass in the Southern U.S. Agric. Ecosyst. Environ. 141, 332–341 (2011).

