# Supplementary material for: The social inefficiency of regulating indirect land use change due to biofuels
Source: Nat Commun. 2017 Jun 26;8:15513. doi: 10.1038/ncomms15513 (PMC5490190; doi:10.1038/ncomms15513)
Supplement: Supplementary Information [file ncomms15513-s1.pdf]

Description of Supplementary Files

File name: Supplementary Information
Description: Supplementary figures, supplementary tables and supplementary references.

Supplementary Table 1. ILUC Factors Estimated by Various Studies

(gCO2e/MJ)	Corn ethanol	Miscanthus ethanol	Switchgrass ethanol	Soybean biodiesel	Sugarcane ethanol	Corn Stover ethanol
**EPA (2010)**[1]	30.3 (19.9-43.6)		14.2 (8.5-21.8)	40.8 (14.2-72)	3.8 (-4.7-11.4)	
**Taheripour and Tyner(2013)**[2]	12.9 (12.9-22.6)	5.8 (5.8-32.3)	20.3 (20.3-74)			-1 (-0.9-1.6)
**CARB (2014)**[3]	19.8			29.1	11.8	
**CARB (2009)**[4]	30 (18.3-44.3)		18	42 (27-51)	46 (32.3-56.7)	
**Hertel et al (2010)**[5]	27 (14.7-90)					
**Tyner et al (2010)**[6]	(14.5-22.9)					
**Searchinger et al (2008)**[7]	104		111			
**Dumortier et al (2009)**[8]	(14-63)					
**Plevin (2010)**[9]	(21-142)					

Supplementary Table 2. Values of Direct Biofuel Carbon Intensity and ILUC Factors Assumed in this Study for Alternative Scenarios

(gCO2e/MJ)	Carbon Intensity	ILUC Factors		
		CARB	EPA	Searchinger
Corn Ethanol	56.7	19.8	30.3	104
Miscanthus Ethanol	-57.2	5.8[1]	14.2[2]	111[2]
Switchgrass Ethanol	-48.1	20.3[1]	14.2	111
Corn Stover Ethanol	25.5	-1 [1]	-1 [1]	-1 [1]
Willow Ethanol[4]	-22.5	0	0	0
Poplar Ethanol[4]	-13.1	0	0	0
Energy Cane Ethanol[4]	-18.1	0	0	0
Soybean Diesel	34.7	29.1	40.8	40.8[3]
Sugarcane Ethanol	26.1	11.8	3.8	3.8[3]

1 Taheripour, F. & Tyner, W. E. Induced land use emissions due to first and second generation biofuels and uncertainty in land use emission factors. *Econ. Res. Int.* **2013,** 1–12 (2013).

2. ILUC factor for miscanthus was not calculated by this study. We therefore assumed it to follow the estimated value of switchgrass

3. ILUC factor was unknown and assumed to be the same as the EPA estimate

4. ILUC factor was unknown and assumed to be zero since these feedstocks are grown in regions of the US that are not major contributors to food and feed production.

Supplementary Table 3: Biofuel Production and Land Use under Alternative Policy Scenarios in 2027

	No_LCFS	LCFS_NO_ILUC Factor	LCFS_With_ILUC Factor		
			CARB	EPA	Searchinger
**Billion Liters of Biofuel**					
**Corn Ethanol**	56.8	19.3	1.6	0	0
**Crop Residue Ethanol**	47.2	2.5	13.2	7.2	49.3
**Perennial Grass Ethanol**	23.5	107.9	116.4	125.5	80.4
**Advanced Biofuel (Sugarcane and Biodiesel)**	7.7	6.2	4.9	4.0	9.3
**Million Hectares**					
**Land Under Crop Residues**	28.5	2.0	8.7	4.1	30.1
**Land Under Energy Crops**	4.2	23.2	24.0	27.0	30.4
**Land Under Corn for Ethanol**	13.7	4.6	0.5	0.0	0.0
**Land Under Crop Production for Food Crops**	76.7	73.5	76.9	75.4	73.2

Supplementary Table 4. Effects of Alternative Policies on Discounted Value of Economic Surplus, 2007-2027 ($ Billion)

	**No_LCFS**	**LCFS_NO_ILUC Factor**	**LCFS_With_ILUC Factor**		
			CARB	EPA	Searchinger
**Social Welfare ($B)**		**Change Relative to No LCFS**	**Change Relative to LCFS_No_ILUC Factor: Additional Cost of Abatement Due to Inclusion of ILUC Factor** [1]		
Fuel Producer	2259	12	-12	-16	-138
Agricultural and Forestry Producers	1878	-67	-8	-11	58
**Total Producer's Surplus**	**4137**	**-55**	**-20**	**-27**	**-80**
Fuel Consumers	19061	78	-18	-32	-176
Agricultural and Forestry Consumers	3328	11	3	9	45
**Total Consumer's Surplus**	**22389**	**89**	**-15**	**-24**	**-131**
Government Revenue	988	0.5	0.5	0.6	0.6
**Total**	**27514**	**35**	**-35**	**-50**	**-211**

[1] Figures have been rounded to the nearest integer except for Government Revenue. Total values are subject to rounding error. Positive numbers represent a gain in economic surplus while negative numbers represent a loss. Estimates are obtained with a 3% discount rate.

Supplementary Figure 1. Policy Targets Assumed in this Study

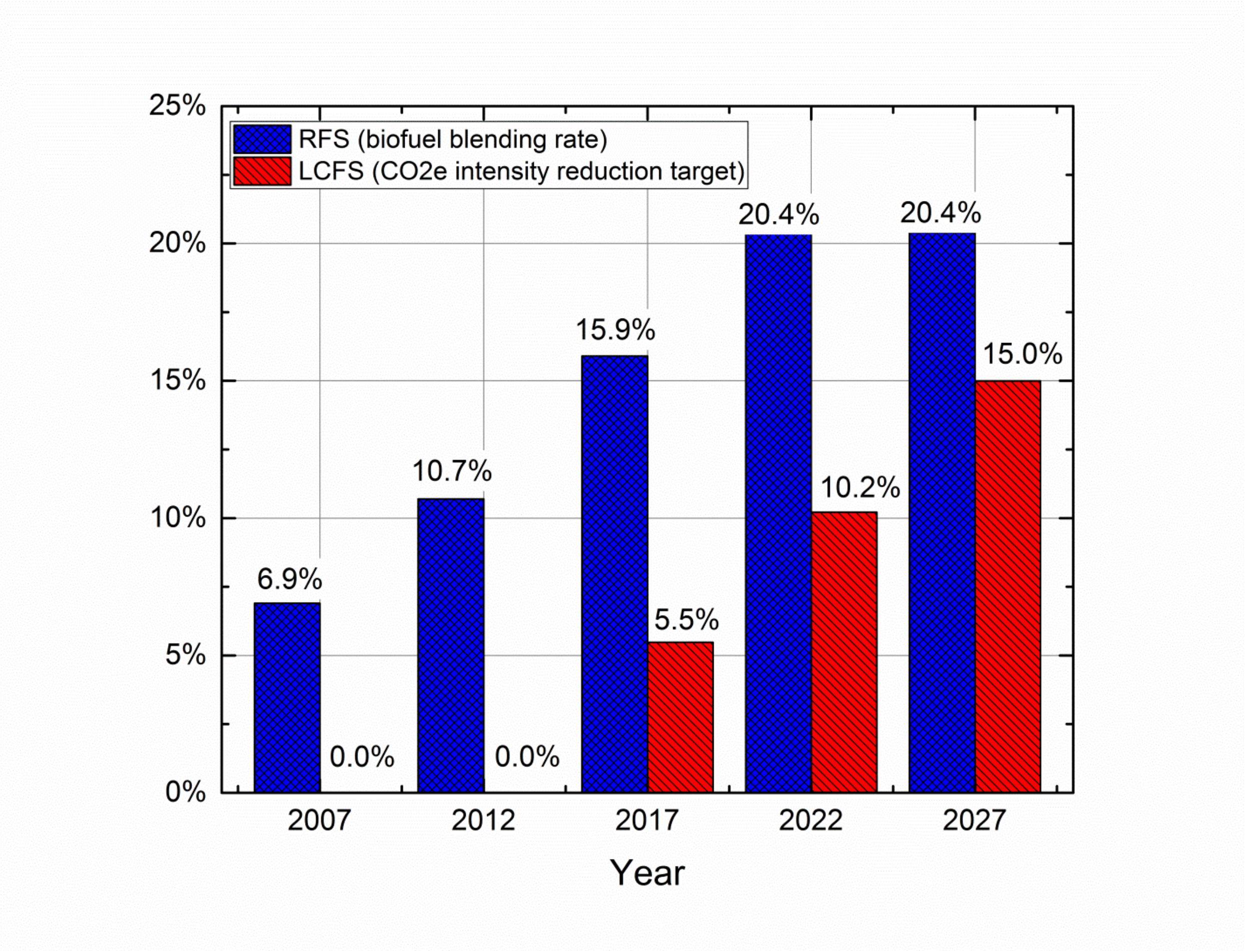

The LCFS targets are defined as targeted reductions in average carbon intensity of transportation fuel relative to a baseline carbon intensity of transportation fuel of 93 g $CO2e$ MJ^{-1} as in[10].

Supplementary Figure 2. Sensitivity Analysis of the Additional US Cost of Abatement per Mg CO_2e Due to the Inclusion of the ILUC Factor

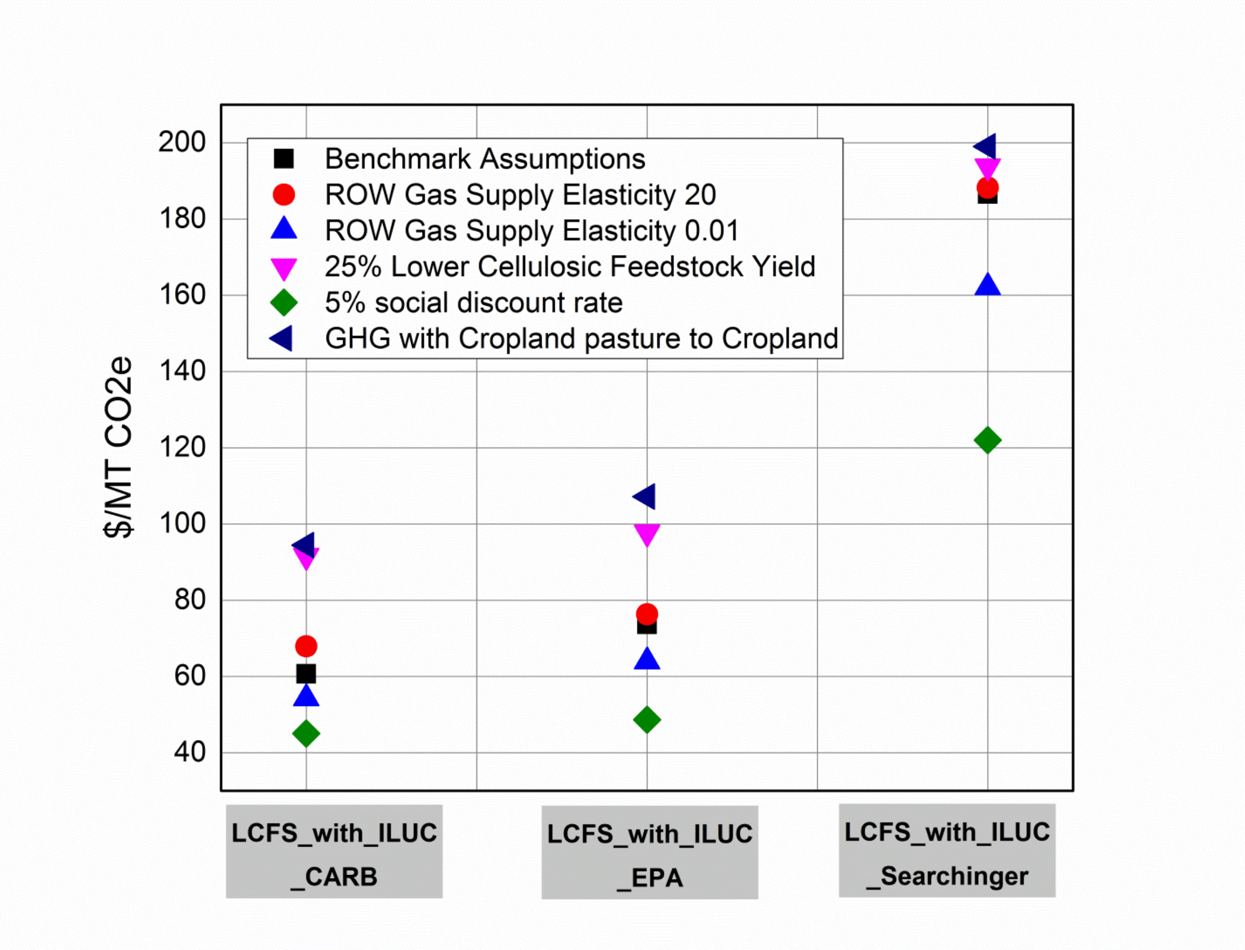

Supplementary References

1. Environmental Protection Agency. *Renewable Fuel Standard Program (RFS2) Regulatory Impact Analysis Program.* (doi:EPA-420-R-10-006, 2010).
2. Taheripour, F. & Tyner, W. E. Induced land use emissions due to first and second generation biofuels and uncertainty in land use emission factors. *Econ. Res. Int.* **2013,** 1–12 (2013).
3. California Air Resources Board. *Staff Report: Initial Statement of Reasons for Proposed Rulemaking. Industrial Strategies Division, California Environmental Protection Agency. (2014).* (Available at: http://www.arb.ca.gov/regact/2015/lcfs2015/lcfs15isor.pdf).
4. California Air Resources Board. *Proposed staff report: initial statement of reasons. Regulation to implement the low carbon fuel standard. (2009).* (Available at: https://www.arb.ca.gov/fuels/lcfs/030409lcfs_isor_vol1.pdf).
5. Hertel, T. W. *et al.* Effects of US maize ethanol on global land use and greenhouse gas emissions: estimating market-mediated responses. *BioScience* **60,** 223–231 (2010).
6. Tyner, W., Taheripour, F., Zhuang, Q., Birur, D. & Baldos, U. *Land use changes and consequent CO2 emissions due to US corn ethanol production: A comprehensive analysis.* (West Lafayette, IN, USA: Department of Agricultural Economics, Purdue university, 2010).
7. Searchinger, T. *et al.* Use of U.S. croplands for biofuels increases greenhouse gases through emissions from land-use change. *Science* **319,** 1238–1240 (2008).
8. Dumortier, J. *et al.* Sensitivity of carbon emission estimates from indirect land-use change. *Appl. Econ. Perspect. Policy* **33,** 428–448 (2011).
9. Plevin, R. J., Jones, A. D., Torn, M. S. & Gibbs, H. K. Greenhouse gas emissions from biofuels' indirect land use change are uncertain but may be much greater than previously estimated. *Environ. Sci. Technol.* **44,** 8015–8021 (2010).

10. Chen, X., Huang, H., Khanna, M. & Önal, H. Alternative transportation fuel standards: Welfare effects and climate benefits. *J. Environ. Econ. Manag.* **67,** 241–257 (2014).
